# Timeliness of a potential automated system for national surveillance of healthcare-associated infections in England

**DOI:** 10.1016/j.jhin.2025.04.008

**Published:** 2025-04-25

**Authors:** T. Phuong Quan, David W. Eyre, Stephanie Shadwell, Daniel West, Susan Hopkins, Dimple Chudasama, Berit Muller-Pebody, Russell Hope, A. Sarah Walker

**Affiliations:** 1 Nuffield Department of Medicine, University of Oxford, Oxford, UK; 2 The National Institute for Health Research Health Protection Research Unit in Healthcare Associated Infections and Antimicrobial Resistance at the University of Oxford, Oxford, UK; 3 The National Institute for Health Research Oxford Biomedical Research Centre, University of Oxford, Oxford, UK; 4 Big Data Institute, Nuffield Department of Population Health, University of Oxford, Oxford, UK; 5 Data, Analytics and Surveillance Group, UK Health Security Agency, London, UK; 6 Clinical and Public Health Group, UK Health Security Agency, London, UK

Surveillance of healthcare-associated infections (HCAIs) is important for public health, however data collection and reporting can be burdensome for healthcare staff[1]. In England, local hospital groups are required to submit their HCAI cases every month to the UK Health Security Agency (UKHSA) via a web-based “Data Capture System” portal[2].

The aim of this study was to assess the timeliness of data points that could be used for a centrally-implemented, automated HCAI surveillance system in England, as a potential alternative to the current locally-implemented system.

We set up prospective daily monitoring of existing UKHSA data feeds relevant to HCAI surveillance. Microbiology data was obtained directly from regional laboratories, covering infection episodes and antimicrobial susceptibility tests for a range of bloodstream infections of differing prevalence including those subject to mandatory surveillance (*E. coli, S. aureus, K. pneumoniae, Enterococcus spp*., *Pseudomonas spp*., *S. pneumoniae, K. oxytoca, Acinetobacter spp*.); patient data was obtained via the Secondary Uses Service (SUS+)[3], covering emergency department (and urgent care) attendances, plus inpatient admission, discharge, and diagnosis codes. We monitored numbers of records available for seven arbitrary activity dates (between 14 Nov 2022 and 1 Sep 2023), every day for at least six months. For each activity date, we calculated the number of days it took to receive 50/90/99 percent of the records relating to that date, and the number of days it took to receive the first/last record from 50/90/99 percent of organisations. See Supplementary material for further details.

At the end of the study period (22 Mar 2024) there were 233-305 infection episode records and 3133-4136 susceptibility records across the 7 activity dates, from 104 and 98 laboratories respectively. For patient data, there were 61,746-80,533 emergency department records and 31,560-63,326 inpatient records, from 492 and 191 providers respectively.

It took 1-3 weeks for 90% of bloodstream infection records pertaining to specimens collected on a particular date to become available, up to a month for 90% of relevant admission/emergency department dates (relevant for determining HCAI onset as community vs hospital-acquired), and up to two months for 90% of inpatient diagnosis codes (relevant for determining risk factors), see Figure 1. It sometimes took a year for final records to arrive, for unclear reasons.

Receipt of inpatient data appeared to be highly organisation-specific, with 10% of providers taking up to 6 months to complete their submissions. In addition, 64-72% of providers did not submit a record until a discharge date was present, and 30-41% of providers did not submit a record until both a discharge date and diagnosis codes were present.

While new microbiology and patient records are received and loaded at UKHSA every day, this does not mean that all the records relating to a particular activity date will arrive the following day. This is likely because many organisations send their data in batches rather than in daily instalments. This means that implementing HCAI surveillance centrally at UKHSA would result in slower ascertainment than the present system, where hospital groups submit their monthly cases by the 15^th^ day after the month end. However, it is important to recognise that this current level of timeliness reflects current practice rather than a hard limit. For comparison, during the first pandemic wave of COVID-19 in England, around 85% of laboratory-confirmed cases of COVID-19 were available at UKHSA within 4 days of specimen collection[4].

Answering the question “how fast is fast enough?” depends on what the data will be used for. Slower ascertainment will affect outbreak identification, in which case it may be worth investing in improving feeds from the slowest organisations. Alternatively, if long-term trends or regional variations are the focus, timeliness may be less important than the consistency of implementation across different hospitals, which is more easily achievable with centrally-based surveillance.

One study limitation is that it only tracked total numbers of records on each day, and did not track changes to records that had been loaded on previous days. It is not unexpected for data to be updated after initial receipt, whether due to factors occurring at the originating source or factors relating to the loading process. This means it will also be necessary to establish how long the data should be treated as provisional and when it can be considered to be stable and “complete”. Furthermore, automated processes need regular monitoring and maintenance to ensure that they are functioning as expected and to avoid missing data. Whilst our study was conducted only in England, the issues it raises are likely generalisable across other countries considering centrally-implemented surveillance[5].

HCAI surveillance is a labour-intensive process and so automation is a desirable goal, but automation at a national level is still rare[5]. Understanding the capabilities as well as the limitations of automated data feeds is essential for ensuring that any resulting system will meet the needs and expectations of its users.

## Supplementary Material

Supplementary material

## Figures and Tables

**Figure 1:**
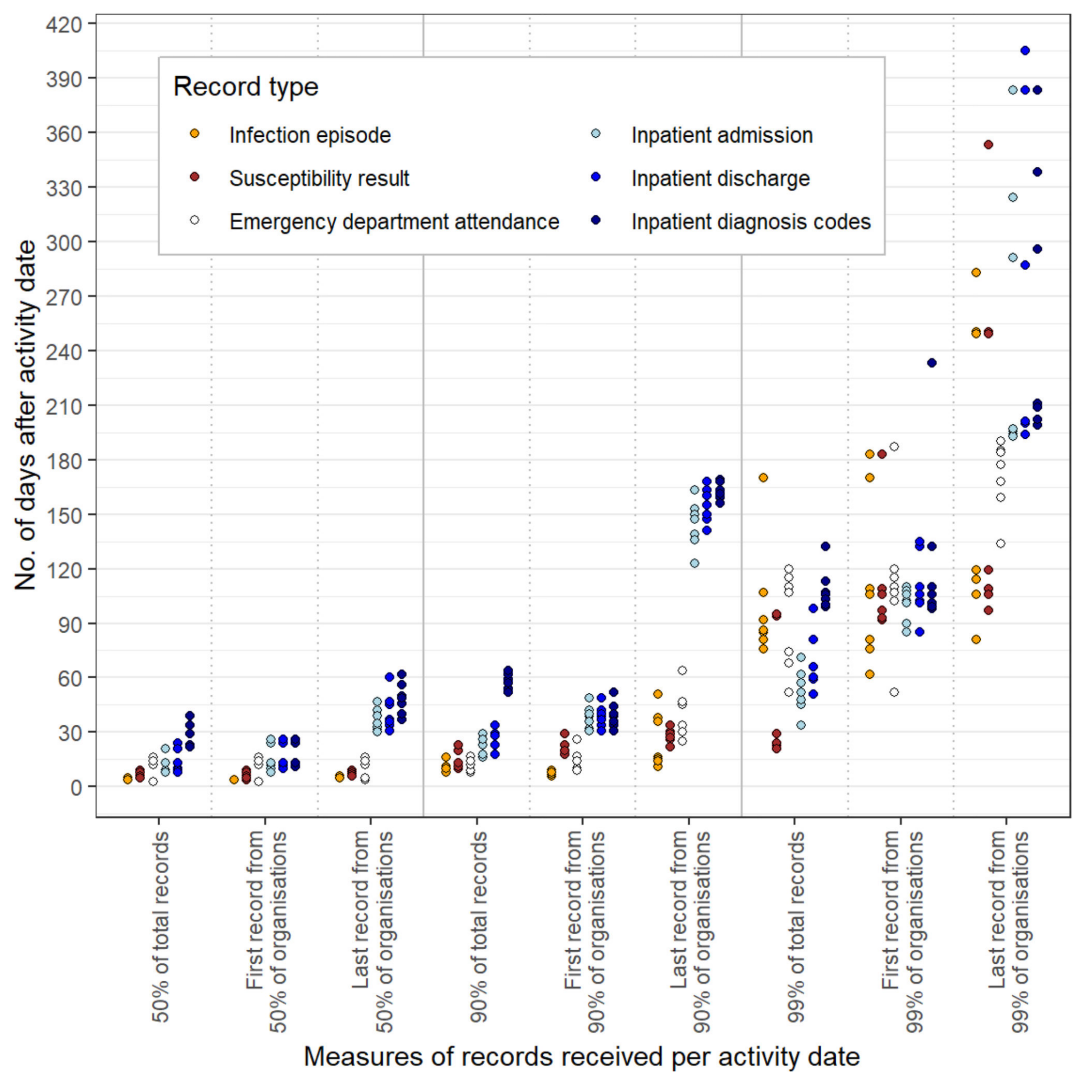
Time to availability of different types of data records relevant for HCAI surveillance, across all seven activity dates.
