# Supplementary material for: Timeliness of a potential automated system for national surveillance of healthcare-associated infections in England
Source: J Hosp Infect. Author manuscript; Available in PMC 2026 Mar 11. (PMC7618852; doi:10.1016/j.jhin.2025.04.008)
Supplement: Supplementary material [file EMS212846-supplement-Supplementary_material.docx]

**Timeliness of a potential automated system for national surveillance of healthcare-associated infections in England – Supplementary material**

**ADDITIONAL METHODS**

**Microbiology data**

The Second Generation Surveillance System (SGSS) is a data warehouse operated by UKHSA which receives test results from the vast majority of microbiology laboratories in England. It is updated daily and contains two distinct modules (which are populated from separate data feeds):

- **CDR** – this contains microbiologically-confirmed infection episodes, deduplicated to a e.g. 14 or 28-day window depending on the organism
- **AMR** – this contains individual antimicrobial susceptibility test results, including both resistant and susceptible results

**Patient data**

The Secondary Uses Service (SUS+)[1] is a data warehouse operated by NHS England which contains patient-level information submitted by secondary care providers via a number of Commissioning Data Sets[2]. Among the datasets which are updated and provided to UKHSA on a daily basis are:

- **APC** - The Admitted Patient Care dataset contains data regarding inpatient admissions at both NHS and independent sector providers. It includes diagnosis codes which describe the primary condition the patient was treated for, and any other secondary condition(s) and relevant comorbidities.
- **ECDS** – the Emergency Care Data Set contains data regarding emergency department and urgent care attendances.

Note, this data source is not to be confused with Hospital Episode Statistics (HES)[3], which is a downstream product of SUS+ and is only produced monthly by NHS England.

**Analysis methods**

For each of seven activity dates (14 Nov 2022, 6 Dec 2022, 9 Jan 2023, 19/24/29 Aug 2023, 1 Sep 2023), we created time series of the numbers of records that were available on each subsequent day, followed for at least 6 months (examples for activity date 14 Nov 2022 are shown in Supplementary Figure S1). The activity dates were chosen arbitrarily, and were roughly spread across days of the week, however noting that the small number of dates followed would mean that no inference could be made into any particular day of the week. There was no specific intention to exclude Wednesday or Sunday. The percentage of total records available on each date was calculated as the number of records received by that day divided by the total number of records received at the end of the study period (22 Mar 2024). The percentage of organisations submitting their first/last record on a particular day was calculated as the number of organisations whose first/last record was received by that day divided by the total number of organisations submitting that record type during the study period.

For microbiology data we counted the number of (CDR) infection episode records and the number of (AMR) susceptibility records with a particular index specimen date, for a range of bloodstream infections of differing prevalence (*E. coli*, *S. aureus*, *K. pneumoniae*, *Enterococcus spp.*, *Pseudomonas spp*., *S. pneumoniae*, *K. oxytoca*, *Acinetobacter spp.*). We also counted the number of laboratories that had reported at least one of the above infections for that specimen date by the end of the study period.

For patient data we counted the number of (APC) inpatient admissions (i.e. the number admitted, number discharged, and number discharged with one or more diagnosis codes available) and the number of (ECDS) emergency department attendances commencing on a particular activity date. We also counted the number of providers that had reported at least one record for that activity date. Outpatient attendances were not considered.

**Data and code availability**

The dataset generated and analysed for this study is available from the Zenodo repository, [https://zenodo.org/doi/10.5281/zenodo.12805820]. All names of laboratories and health providers have been anonymised. All analyses were conducted using R v4.3.1, and the code is openly available from Zenodo [https://zenodo.org/doi/10.5281/zenodo.13354506] and GitHub [https://github.com/oxfordmmm/ukhsa-datafeeds-timeliness-anon].

**ADDITIONAL RESULTS**

**Microbiology data**

50% of the microbiology records were available within 4-9 days (range across the 7 activity dates), 90% within 8-23 days, and 99% within 21-170 days (Figure 1, Table S1). Of all the laboratories that submitted at least one record, 50% sent their first (last) record within 4-9 (5-9) days, 90% within 6-29 (11-51) days, and 99% within 62-183 (81-353) days.

Records relating to rarer organisms like *Acinetobacter spp.* were more variable with 90% of records available within 6-183 days, whereas for *E. coli* 90% of records were available within 7-29 days after the activity date.

**Patient data**

There was a period of disruption in the loading of APC and ECDS records in August and September 2023, with delays to loading and some temporary instability in the cumulative numbers of records. These activity dates have been included in the analyses but are presented separately below where appropriate.

90% of inpatient admission records, discharge dates, and diagnosis codes, were available within 16-29 days, 18-34 days, and 52-64 days respectively. 50% of providers sent their first (last) record within 8-26 (58-95) days, 90% within 31-49 (85-202) days, and 99% within 85-110 (97-338) days.

For emergency department attendances, due to the loading disruption, 4 of the activity dates (19/24/29 Aug 2023, 1 Sep 2023) had the vast majority of their records bulk loaded on a single day, and so were not representative of the time it took for providers to send their data. For the 3 dates that were not disrupted, 50% of records arrived within 3 days, 90% within 8-9 days, and 99% within 52-74 days. In contrast, for the 4 disrupted activity dates, 50% of the records arrived within 3-16 days, 90% within 12-17 days, and 99% within 107-120 days.

**REFERENCES**

[1] NHS Digital. Secondary Uses Service (SUS) [22 December 2023]. Available from: <https://digital.nhs.uk/services/secondary-uses-service-sus>.

[2] NHS Digital. Commissioning Data Sets [10 April 2024]. Available from: <https://digital.nhs.uk/data-and-information/data-collections-and-data-sets/data-sets/commissioning-data-sets>.

[3] NHS Digital. Hospital Episode Statistics (HES) [05 March 2024]. Available from: <https://digital.nhs.uk/data-and-information/data-tools-and-services/data-services/hospital-episode-statistics>.

**Figure S1.** A selection of time series showing the availability of records for the specimen/admission/arrival date of 14 Nov 2022, on each subsequent day until the end of the study period (22 Mar 2024). **A.** Number of infection episode (CDR) records. **B.** Number of laboratories with at least one susceptibility (AMR) record. **C.** Number of inpatient admission (APC) records. **D.** Number of providers with at least one emergency department (ECDS) record.


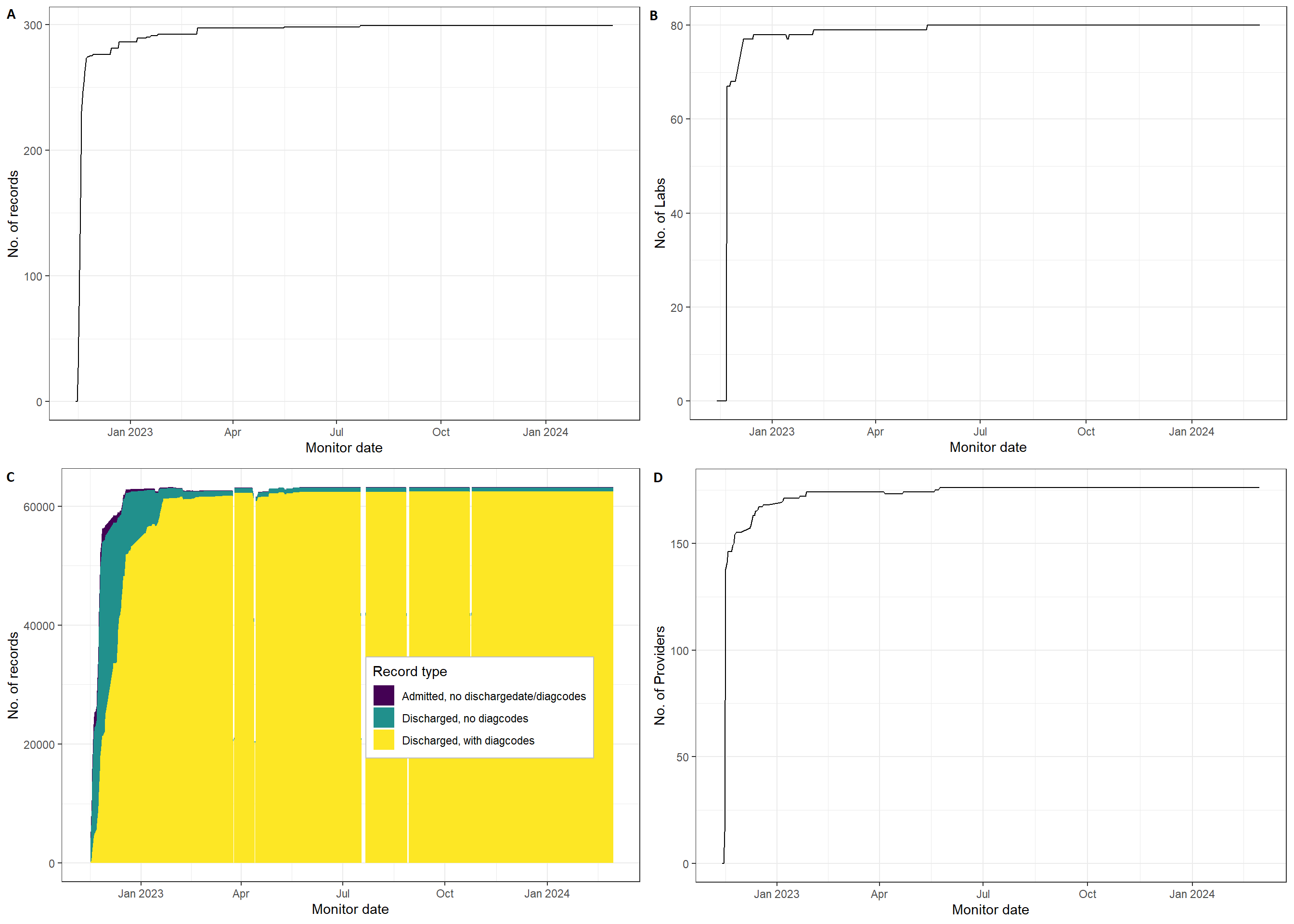


**Table S1.** Time to availability of the different types of data records monitored, for all seven activity dates

| **Activity date** | **Record type** | **Measure** | **Days** |
| --- | --- | --- | --- |
| 2022-11-14 | Infection episode | 50% of total records | 4 |
| 2022-11-14 | Infection episode | 90% of total records | 9 |
| 2022-11-14 | Infection episode | 99% of total records | 107 |
| 2022-11-14 | Infection episode | First record from 50% of organisations | 4 |
| 2022-11-14 | Infection episode | First record from 90% of organisations | 6 |
| 2022-11-14 | Infection episode | First record from 99% of organisations | 62 |
| 2022-11-14 | Infection episode | Last record from 50% of organisations | 5 |
| 2022-11-14 | Infection episode | Last record from 90% of organisations | 38 |
| 2022-11-14 | Infection episode | Last record from 99% of organisations | 250 |
| 2022-11-14 | Susceptibility result | 50% of total records | 9 |
| 2022-11-14 | Susceptibility result | 90% of total records | 10 |
| 2022-11-14 | Susceptibility result | 99% of total records | 23 |
| 2022-11-14 | Susceptibility result | First record from 50% of organisations | 9 |
| 2022-11-14 | Susceptibility result | First record from 90% of organisations | 23 |
| 2022-11-14 | Susceptibility result | First record from 99% of organisations | 183 |
| 2022-11-14 | Susceptibility result | Last record from 50% of organisations | 9 |
| 2022-11-14 | Susceptibility result | Last record from 90% of organisations | 30 |
| 2022-11-14 | Susceptibility result | Last record from 99% of organisations | 250 |
| 2022-11-14 | Emergency department attendance | 50% of total records | 3 |
| 2022-11-14 | Emergency department attendance | 90% of total records | 9 |
| 2022-11-14 | Emergency department attendance | 99% of total records | 74 |
| 2022-11-14 | Emergency department attendance | First record from 50% of organisations | 3 |
| 2022-11-14 | Emergency department attendance | First record from 90% of organisations | 26 |
| 2022-11-14 | Emergency department attendance | First record from 99% of organisations | 187 |
| 2022-11-14 | Emergency department attendance | Last record from 50% of organisations | 5 |
| 2022-11-14 | Emergency department attendance | Last record from 90% of organisations | 64 |
| 2022-11-14 | Emergency department attendance | Last record from 99% of organisations | 190 |
| 2022-11-14 | Inpatient admission | 50% of total records | 9 |
| 2022-11-14 | Inpatient admission | 90% of total records | 23 |
| 2022-11-14 | Inpatient admission | 99% of total records | 34 |
| 2022-11-14 | Inpatient admission | First record from 50% of organisations | 10 |
| 2022-11-14 | Inpatient admission | First record from 90% of organisations | 32 |
| 2022-11-14 | Inpatient admission | First record from 99% of organisations | 90 |
| 2022-11-14 | Inpatient admission | Last record from 50% of organisations | 32 |
| 2022-11-14 | Inpatient admission | Last record from 90% of organisations | 153 |
| 2022-11-14 | Inpatient admission | Last record from 99% of organisations | 291 |
| 2022-11-14 | Inpatient discharge | 50% of total records | 10 |
| 2022-11-14 | Inpatient discharge | 90% of total records | 23 |
| 2022-11-14 | Inpatient discharge | 99% of total records | 51 |
| 2022-11-14 | Inpatient discharge | First record from 50% of organisations | 10 |
| 2022-11-14 | Inpatient discharge | First record from 90% of organisations | 34 |
| 2022-11-14 | Inpatient discharge | First record from 99% of organisations | 132 |
| 2022-11-14 | Inpatient discharge | Last record from 50% of organisations | 34 |
| 2022-11-14 | Inpatient discharge | Last record from 90% of organisations | 155 |
| 2022-11-14 | Inpatient discharge | Last record from 99% of organisations | 405 |
| 2022-11-14 | Inpatient diagnosis codes | 50% of total records | 23 |
| 2022-11-14 | Inpatient diagnosis codes | 90% of total records | 53 |
| 2022-11-14 | Inpatient diagnosis codes | 99% of total records | 132 |
| 2022-11-14 | Inpatient diagnosis codes | First record from 50% of organisations | 11 |
| 2022-11-14 | Inpatient diagnosis codes | First record from 90% of organisations | 34 |
| 2022-11-14 | Inpatient diagnosis codes | First record from 99% of organisations | 132 |
| 2022-11-14 | Inpatient diagnosis codes | Last record from 50% of organisations | 37 |
| 2022-11-14 | Inpatient diagnosis codes | Last record from 90% of organisations | 163 |
| 2022-11-14 | Inpatient diagnosis codes | Last record from 99% of organisations | 338 |
| 2022-12-06 | Infection episode | 50% of total records | 4 |
| 2022-12-06 | Infection episode | 90% of total records | 16 |
| 2022-12-06 | Infection episode | 99% of total records | 85 |
| 2022-12-06 | Infection episode | First record from 50% of organisations | 4 |
| 2022-12-06 | Infection episode | First record from 90% of organisations | 9 |
| 2022-12-06 | Infection episode | First record from 99% of organisations | 183 |
| 2022-12-06 | Infection episode | Last record from 50% of organisations | 5 |
| 2022-12-06 | Infection episode | Last record from 90% of organisations | 36 |
| 2022-12-06 | Infection episode | Last record from 99% of organisations | 283 |
| 2022-12-06 | Susceptibility result | 50% of total records | 8 |
| 2022-12-06 | Susceptibility result | 90% of total records | 10 |
| 2022-12-06 | Susceptibility result | 99% of total records | 29 |
| 2022-12-06 | Susceptibility result | First record from 50% of organisations | 8 |
| 2022-12-06 | Susceptibility result | First record from 90% of organisations | 29 |
| 2022-12-06 | Susceptibility result | First record from 99% of organisations | 92 |
| 2022-12-06 | Susceptibility result | Last record from 50% of organisations | 8 |
| 2022-12-06 | Susceptibility result | Last record from 90% of organisations | 29 |
| 2022-12-06 | Susceptibility result | Last record from 99% of organisations | 353 |
| 2022-12-06 | Emergency department attendance | 50% of total records | 3 |
| 2022-12-06 | Emergency department attendance | 90% of total records | 8 |
| 2022-12-06 | Emergency department attendance | 99% of total records | 52 |
| 2022-12-06 | Emergency department attendance | First record from 50% of organisations | 3 |
| 2022-12-06 | Emergency department attendance | First record from 90% of organisations | 10 |
| 2022-12-06 | Emergency department attendance | First record from 99% of organisations | 52 |
| 2022-12-06 | Emergency department attendance | Last record from 50% of organisations | 4 |
| 2022-12-06 | Emergency department attendance | Last record from 90% of organisations | 45 |
| 2022-12-06 | Emergency department attendance | Last record from 99% of organisations | 168 |
| 2022-12-06 | Inpatient admission | 50% of total records | 9 |
| 2022-12-06 | Inpatient admission | 90% of total records | 29 |
| 2022-12-06 | Inpatient admission | 99% of total records | 45 |
| 2022-12-06 | Inpatient admission | First record from 50% of organisations | 12 |
| 2022-12-06 | Inpatient admission | First record from 90% of organisations | 42 |
| 2022-12-06 | Inpatient admission | First record from 99% of organisations | 110 |
| 2022-12-06 | Inpatient admission | Last record from 50% of organisations | 42 |
| 2022-12-06 | Inpatient admission | Last record from 90% of organisations | 163 |
| 2022-12-06 | Inpatient admission | Last record from 99% of organisations | 383 |
| 2022-12-06 | Inpatient discharge | 50% of total records | 9 |
| 2022-12-06 | Inpatient discharge | 90% of total records | 29 |
| 2022-12-06 | Inpatient discharge | 99% of total records | 51 |
| 2022-12-06 | Inpatient discharge | First record from 50% of organisations | 12 |
| 2022-12-06 | Inpatient discharge | First record from 90% of organisations | 42 |
| 2022-12-06 | Inpatient discharge | First record from 99% of organisations | 110 |
| 2022-12-06 | Inpatient discharge | Last record from 50% of organisations | 45 |
| 2022-12-06 | Inpatient discharge | Last record from 90% of organisations | 163 |
| 2022-12-06 | Inpatient discharge | Last record from 99% of organisations | 383 |
| 2022-12-06 | Inpatient diagnosis codes | 50% of total records | 29 |
| 2022-12-06 | Inpatient diagnosis codes | 90% of total records | 59 |
| 2022-12-06 | Inpatient diagnosis codes | 99% of total records | 99 |
| 2022-12-06 | Inpatient diagnosis codes | First record from 50% of organisations | 12 |
| 2022-12-06 | Inpatient diagnosis codes | First record from 90% of organisations | 44 |
| 2022-12-06 | Inpatient diagnosis codes | First record from 99% of organisations | 110 |
| 2022-12-06 | Inpatient diagnosis codes | Last record from 50% of organisations | 46 |
| 2022-12-06 | Inpatient diagnosis codes | Last record from 90% of organisations | 169 |
| 2022-12-06 | Inpatient diagnosis codes | Last record from 99% of organisations | 383 |
| 2023-01-09 | Infection episode | 50% of total records | 4 |
| 2023-01-09 | Infection episode | 90% of total records | 11 |
| 2023-01-09 | Infection episode | 99% of total records | 170 |
| 2023-01-09 | Infection episode | First record from 50% of organisations | 4 |
| 2023-01-09 | Infection episode | First record from 90% of organisations | 8 |
| 2023-01-09 | Infection episode | First record from 99% of organisations | 170 |
| 2023-01-09 | Infection episode | Last record from 50% of organisations | 5 |
| 2023-01-09 | Infection episode | Last record from 90% of organisations | 51 |
| 2023-01-09 | Infection episode | Last record from 99% of organisations | 249 |
| 2023-01-09 | Susceptibility result | 50% of total records | 5 |
| 2023-01-09 | Susceptibility result | 90% of total records | 11 |
| 2023-01-09 | Susceptibility result | 99% of total records | 24 |
| 2023-01-09 | Susceptibility result | First record from 50% of organisations | 4 |
| 2023-01-09 | Susceptibility result | First record from 90% of organisations | 18 |
| 2023-01-09 | Susceptibility result | First record from 99% of organisations | 93 |
| 2023-01-09 | Susceptibility result | Last record from 50% of organisations | 6 |
| 2023-01-09 | Susceptibility result | Last record from 90% of organisations | 26 |
| 2023-01-09 | Susceptibility result | Last record from 99% of organisations | 249 |
| 2023-01-09 | Emergency department attendance | 50% of total records | 3 |
| 2023-01-09 | Emergency department attendance | 90% of total records | 9 |
| 2023-01-09 | Emergency department attendance | 99% of total records | 68 |
| 2023-01-09 | Emergency department attendance | First record from 50% of organisations | 3 |
| 2023-01-09 | Emergency department attendance | First record from 90% of organisations | 9 |
| 2023-01-09 | Emergency department attendance | First record from 99% of organisations | 102 |
| 2023-01-09 | Emergency department attendance | Last record from 50% of organisations | 5 |
| 2023-01-09 | Emergency department attendance | Last record from 90% of organisations | 47 |
| 2023-01-09 | Emergency department attendance | Last record from 99% of organisations | 134 |
| 2023-01-09 | Inpatient admission | 50% of total records | 9 |
| 2023-01-09 | Inpatient admission | 90% of total records | 16 |
| 2023-01-09 | Inpatient admission | 99% of total records | 48 |
| 2023-01-09 | Inpatient admission | First record from 50% of organisations | 10 |
| 2023-01-09 | Inpatient admission | First record from 90% of organisations | 39 |
| 2023-01-09 | Inpatient admission | First record from 99% of organisations | 108 |
| 2023-01-09 | Inpatient admission | Last record from 50% of organisations | 33 |
| 2023-01-09 | Inpatient admission | Last record from 90% of organisations | 150 |
| 2023-01-09 | Inpatient admission | Last record from 99% of organisations | 324 |
| 2023-01-09 | Inpatient discharge | 50% of total records | 9 |
| 2023-01-09 | Inpatient discharge | 90% of total records | 18 |
| 2023-01-09 | Inpatient discharge | 99% of total records | 59 |
| 2023-01-09 | Inpatient discharge | First record from 50% of organisations | 10 |
| 2023-01-09 | Inpatient discharge | First record from 90% of organisations | 39 |
| 2023-01-09 | Inpatient discharge | First record from 99% of organisations | 135 |
| 2023-01-09 | Inpatient discharge | Last record from 50% of organisations | 37 |
| 2023-01-09 | Inpatient discharge | Last record from 90% of organisations | 168 |
| 2023-01-09 | Inpatient discharge | Last record from 99% of organisations | 287 |
| 2023-01-09 | Inpatient diagnosis codes | 50% of total records | 22 |
| 2023-01-09 | Inpatient diagnosis codes | 90% of total records | 54 |
| 2023-01-09 | Inpatient diagnosis codes | 99% of total records | 107 |
| 2023-01-09 | Inpatient diagnosis codes | First record from 50% of organisations | 11 |
| 2023-01-09 | Inpatient diagnosis codes | First record from 90% of organisations | 39 |
| 2023-01-09 | Inpatient diagnosis codes | First record from 99% of organisations | 233 |
| 2023-01-09 | Inpatient diagnosis codes | Last record from 50% of organisations | 40 |
| 2023-01-09 | Inpatient diagnosis codes | Last record from 90% of organisations | 168 |
| 2023-01-09 | Inpatient diagnosis codes | Last record from 99% of organisations | 296 |
| 2023-08-19 | Infection episode | 50% of total records | 4 |
| 2023-08-19 | Infection episode | 90% of total records | 8 |
| 2023-08-19 | Infection episode | 99% of total records | 81 |
| 2023-08-19 | Infection episode | First record from 50% of organisations | 4 |
| 2023-08-19 | Infection episode | First record from 90% of organisations | 6 |
| 2023-08-19 | Infection episode | First record from 99% of organisations | 81 |
| 2023-08-19 | Infection episode | Last record from 50% of organisations | 5 |
| 2023-08-19 | Infection episode | Last record from 90% of organisations | 11 |
| 2023-08-19 | Infection episode | Last record from 99% of organisations | 81 |
| 2023-08-19 | Susceptibility result | 50% of total records | 5 |
| 2023-08-19 | Susceptibility result | 90% of total records | 11 |
| 2023-08-19 | Susceptibility result | 99% of total records | 21 |
| 2023-08-19 | Susceptibility result | First record from 50% of organisations | 4 |
| 2023-08-19 | Susceptibility result | First record from 90% of organisations | 18 |
| 2023-08-19 | Susceptibility result | First record from 99% of organisations | 97 |
| 2023-08-19 | Susceptibility result | Last record from 50% of organisations | 7 |
| 2023-08-19 | Susceptibility result | Last record from 90% of organisations | 22 |
| 2023-08-19 | Susceptibility result | Last record from 99% of organisations | 97 |
| 2023-08-19 | Emergency department attendance | 50% of total records | 3 |
| 2023-08-19 | Emergency department attendance | 90% of total records | 14 |
| 2023-08-19 | Emergency department attendance | 99% of total records | 120 |
| 2023-08-19 | Emergency department attendance | First record from 50% of organisations | 3 |
| 2023-08-19 | Emergency department attendance | First record from 90% of organisations | 17 |
| 2023-08-19 | Emergency department attendance | First record from 99% of organisations | 120 |
| 2023-08-19 | Emergency department attendance | Last record from 50% of organisations | 14 |
| 2023-08-19 | Emergency department attendance | Last record from 90% of organisations | 34 |
| 2023-08-19 | Emergency department attendance | Last record from 99% of organisations | 159 |
| 2023-08-19 | Inpatient admission | 50% of total records | 13 |
| 2023-08-19 | Inpatient admission | 90% of total records | 18 |
| 2023-08-19 | Inpatient admission | 99% of total records | 71 |
| 2023-08-19 | Inpatient admission | First record from 50% of organisations | 13 |
| 2023-08-19 | Inpatient admission | First record from 90% of organisations | 40 |
| 2023-08-19 | Inpatient admission | First record from 99% of organisations | 102 |
| 2023-08-19 | Inpatient admission | Last record from 50% of organisations | 39 |
| 2023-08-19 | Inpatient admission | Last record from 90% of organisations | 139 |
| 2023-08-19 | Inpatient admission | Last record from 99% of organisations | 194 |
| 2023-08-19 | Inpatient discharge | 50% of total records | 13 |
| 2023-08-19 | Inpatient discharge | 90% of total records | 34 |
| 2023-08-19 | Inpatient discharge | 99% of total records | 81 |
| 2023-08-19 | Inpatient discharge | First record from 50% of organisations | 13 |
| 2023-08-19 | Inpatient discharge | First record from 90% of organisations | 40 |
| 2023-08-19 | Inpatient discharge | First record from 99% of organisations | 102 |
| 2023-08-19 | Inpatient discharge | Last record from 50% of organisations | 60 |
| 2023-08-19 | Inpatient discharge | Last record from 90% of organisations | 147 |
| 2023-08-19 | Inpatient discharge | Last record from 99% of organisations | 200 |
| 2023-08-19 | Inpatient diagnosis codes | 50% of total records | 39 |
| 2023-08-19 | Inpatient diagnosis codes | 90% of total records | 62 |
| 2023-08-19 | Inpatient diagnosis codes | 99% of total records | 113 |
| 2023-08-19 | Inpatient diagnosis codes | First record from 50% of organisations | 13 |
| 2023-08-19 | Inpatient diagnosis codes | First record from 90% of organisations | 40 |
| 2023-08-19 | Inpatient diagnosis codes | First record from 99% of organisations | 98 |
| 2023-08-19 | Inpatient diagnosis codes | Last record from 50% of organisations | 62 |
| 2023-08-19 | Inpatient diagnosis codes | Last record from 90% of organisations | 159 |
| 2023-08-19 | Inpatient diagnosis codes | Last record from 99% of organisations | 211 |
| 2023-08-24 | Infection episode | 50% of total records | 5 |
| 2023-08-24 | Infection episode | 90% of total records | 10 |
| 2023-08-24 | Infection episode | 99% of total records | 76 |
| 2023-08-24 | Infection episode | First record from 50% of organisations | 4 |
| 2023-08-24 | Infection episode | First record from 90% of organisations | 7 |
| 2023-08-24 | Infection episode | First record from 99% of organisations | 76 |
| 2023-08-24 | Infection episode | Last record from 50% of organisations | 6 |
| 2023-08-24 | Infection episode | Last record from 90% of organisations | 16 |
| 2023-08-24 | Infection episode | Last record from 99% of organisations | 119 |
| 2023-08-24 | Susceptibility result | 50% of total records | 6 |
| 2023-08-24 | Susceptibility result | 90% of total records | 13 |
| 2023-08-24 | Susceptibility result | 99% of total records | 94 |
| 2023-08-24 | Susceptibility result | First record from 50% of organisations | 6 |
| 2023-08-24 | Susceptibility result | First record from 90% of organisations | 20 |
| 2023-08-24 | Susceptibility result | First record from 99% of organisations | 93 |
| 2023-08-24 | Susceptibility result | Last record from 50% of organisations | 7 |
| 2023-08-24 | Susceptibility result | Last record from 90% of organisations | 34 |
| 2023-08-24 | Susceptibility result | Last record from 99% of organisations | 119 |
| 2023-08-24 | Emergency department attendance | 50% of total records | 12 |
| 2023-08-24 | Emergency department attendance | 90% of total records | 12 |
| 2023-08-24 | Emergency department attendance | 99% of total records | 115 |
| 2023-08-24 | Emergency department attendance | First record from 50% of organisations | 12 |
| 2023-08-24 | Emergency department attendance | First record from 90% of organisations | 14 |
| 2023-08-24 | Emergency department attendance | First record from 99% of organisations | 115 |
| 2023-08-24 | Emergency department attendance | Last record from 50% of organisations | 12 |
| 2023-08-24 | Emergency department attendance | Last record from 90% of organisations | 30 |
| 2023-08-24 | Emergency department attendance | Last record from 99% of organisations | 185 |
| 2023-08-24 | Inpatient admission | 50% of total records | 8 |
| 2023-08-24 | Inpatient admission | 90% of total records | 29 |
| 2023-08-24 | Inpatient admission | 99% of total records | 57 |
| 2023-08-24 | Inpatient admission | First record from 50% of organisations | 8 |
| 2023-08-24 | Inpatient admission | First record from 90% of organisations | 36 |
| 2023-08-24 | Inpatient admission | First record from 99% of organisations | 106 |
| 2023-08-24 | Inpatient admission | Last record from 50% of organisations | 35 |
| 2023-08-24 | Inpatient admission | Last record from 90% of organisations | 123 |
| 2023-08-24 | Inpatient admission | Last record from 99% of organisations | 196 |
| 2023-08-24 | Inpatient discharge | 50% of total records | 8 |
| 2023-08-24 | Inpatient discharge | 90% of total records | 34 |
| 2023-08-24 | Inpatient discharge | 99% of total records | 60 |
| 2023-08-24 | Inpatient discharge | First record from 50% of organisations | 13 |
| 2023-08-24 | Inpatient discharge | First record from 90% of organisations | 37 |
| 2023-08-24 | Inpatient discharge | First record from 99% of organisations | 106 |
| 2023-08-24 | Inpatient discharge | Last record from 50% of organisations | 36 |
| 2023-08-24 | Inpatient discharge | Last record from 90% of organisations | 141 |
| 2023-08-24 | Inpatient discharge | Last record from 99% of organisations | 201 |
| 2023-08-24 | Inpatient diagnosis codes | 50% of total records | 34 |
| 2023-08-24 | Inpatient diagnosis codes | 90% of total records | 57 |
| 2023-08-24 | Inpatient diagnosis codes | 99% of total records | 106 |
| 2023-08-24 | Inpatient diagnosis codes | First record from 50% of organisations | 13 |
| 2023-08-24 | Inpatient diagnosis codes | First record from 90% of organisations | 36 |
| 2023-08-24 | Inpatient diagnosis codes | First record from 99% of organisations | 106 |
| 2023-08-24 | Inpatient diagnosis codes | Last record from 50% of organisations | 56 |
| 2023-08-24 | Inpatient diagnosis codes | Last record from 90% of organisations | 156 |
| 2023-08-24 | Inpatient diagnosis codes | Last record from 99% of organisations | 209 |
| 2023-08-29 | Infection episode | 50% of total records | 4 |
| 2023-08-29 | Infection episode | 90% of total records | 8 |
| 2023-08-29 | Infection episode | 99% of total records | 86 |
| 2023-08-29 | Infection episode | First record from 50% of organisations | 4 |
| 2023-08-29 | Infection episode | First record from 90% of organisations | 7 |
| 2023-08-29 | Infection episode | First record from 99% of organisations | 109 |
| 2023-08-29 | Infection episode | Last record from 50% of organisations | 5 |
| 2023-08-29 | Infection episode | Last record from 90% of organisations | 15 |
| 2023-08-29 | Infection episode | Last record from 99% of organisations | 114 |
| 2023-08-29 | Susceptibility result | 50% of total records | 5 |
| 2023-08-29 | Susceptibility result | 90% of total records | 20 |
| 2023-08-29 | Susceptibility result | 99% of total records | 94 |
| 2023-08-29 | Susceptibility result | First record from 50% of organisations | 4 |
| 2023-08-29 | Susceptibility result | First record from 90% of organisations | 18 |
| 2023-08-29 | Susceptibility result | First record from 99% of organisations | 109 |
| 2023-08-29 | Susceptibility result | Last record from 50% of organisations | 7 |
| 2023-08-29 | Susceptibility result | Last record from 90% of organisations | 29 |
| 2023-08-29 | Susceptibility result | Last record from 99% of organisations | 109 |
| 2023-08-29 | Emergency department attendance | 50% of total records | 16 |
| 2023-08-29 | Emergency department attendance | 90% of total records | 17 |
| 2023-08-29 | Emergency department attendance | 99% of total records | 110 |
| 2023-08-29 | Emergency department attendance | First record from 50% of organisations | 16 |
| 2023-08-29 | Emergency department attendance | First record from 90% of organisations | 17 |
| 2023-08-29 | Emergency department attendance | First record from 99% of organisations | 110 |
| 2023-08-29 | Emergency department attendance | Last record from 50% of organisations | 16 |
| 2023-08-29 | Emergency department attendance | Last record from 90% of organisations | 25 |
| 2023-08-29 | Emergency department attendance | Last record from 99% of organisations | 184 |
| 2023-08-29 | Inpatient admission | 50% of total records | 8 |
| 2023-08-29 | Inpatient admission | 90% of total records | 29 |
| 2023-08-29 | Inpatient admission | 99% of total records | 52 |
| 2023-08-29 | Inpatient admission | First record from 50% of organisations | 24 |
| 2023-08-29 | Inpatient admission | First record from 90% of organisations | 31 |
| 2023-08-29 | Inpatient admission | First record from 99% of organisations | 101 |
| 2023-08-29 | Inpatient admission | Last record from 50% of organisations | 30 |
| 2023-08-29 | Inpatient admission | Last record from 90% of organisations | 136 |
| 2023-08-29 | Inpatient admission | Last record from 99% of organisations | 193 |
| 2023-08-29 | Inpatient discharge | 50% of total records | 24 |
| 2023-08-29 | Inpatient discharge | 90% of total records | 29 |
| 2023-08-29 | Inpatient discharge | 99% of total records | 66 |
| 2023-08-29 | Inpatient discharge | First record from 50% of organisations | 24 |
| 2023-08-29 | Inpatient discharge | First record from 90% of organisations | 31 |
| 2023-08-29 | Inpatient discharge | First record from 99% of organisations | 101 |
| 2023-08-29 | Inpatient discharge | Last record from 50% of organisations | 31 |
| 2023-08-29 | Inpatient discharge | Last record from 90% of organisations | 150 |
| 2023-08-29 | Inpatient discharge | Last record from 99% of organisations | 194 |
| 2023-08-29 | Inpatient diagnosis codes | 50% of total records | 29 |
| 2023-08-29 | Inpatient diagnosis codes | 90% of total records | 52 |
| 2023-08-29 | Inpatient diagnosis codes | 99% of total records | 103 |
| 2023-08-29 | Inpatient diagnosis codes | First record from 50% of organisations | 24 |
| 2023-08-29 | Inpatient diagnosis codes | First record from 90% of organisations | 31 |
| 2023-08-29 | Inpatient diagnosis codes | First record from 99% of organisations | 101 |
| 2023-08-29 | Inpatient diagnosis codes | Last record from 50% of organisations | 50 |
| 2023-08-29 | Inpatient diagnosis codes | Last record from 90% of organisations | 163 |
| 2023-08-29 | Inpatient diagnosis codes | Last record from 99% of organisations | 199 |
| 2023-09-01 | Infection episode | 50% of total records | 4 |
| 2023-09-01 | Infection episode | 90% of total records | 10 |
| 2023-09-01 | Infection episode | 99% of total records | 92 |
| 2023-09-01 | Infection episode | First record from 50% of organisations | 4 |
| 2023-09-01 | Infection episode | First record from 90% of organisations | 8 |
| 2023-09-01 | Infection episode | First record from 99% of organisations | 106 |
| 2023-09-01 | Infection episode | Last record from 50% of organisations | 5 |
| 2023-09-01 | Infection episode | Last record from 90% of organisations | 14 |
| 2023-09-01 | Infection episode | Last record from 99% of organisations | 106 |
| 2023-09-01 | Susceptibility result | 50% of total records | 5 |
| 2023-09-01 | Susceptibility result | 90% of total records | 23 |
| 2023-09-01 | Susceptibility result | 99% of total records | 95 |
| 2023-09-01 | Susceptibility result | First record from 50% of organisations | 5 |
| 2023-09-01 | Susceptibility result | First record from 90% of organisations | 20 |
| 2023-09-01 | Susceptibility result | First record from 99% of organisations | 106 |
| 2023-09-01 | Susceptibility result | Last record from 50% of organisations | 6 |
| 2023-09-01 | Susceptibility result | Last record from 90% of organisations | 27 |
| 2023-09-01 | Susceptibility result | Last record from 99% of organisations | 106 |
| 2023-09-01 | Emergency department attendance | 50% of total records | 14 |
| 2023-09-01 | Emergency department attendance | 90% of total records | 14 |
| 2023-09-01 | Emergency department attendance | 99% of total records | 107 |
| 2023-09-01 | Emergency department attendance | First record from 50% of organisations | 14 |
| 2023-09-01 | Emergency department attendance | First record from 90% of organisations | 14 |
| 2023-09-01 | Emergency department attendance | First record from 99% of organisations | 107 |
| 2023-09-01 | Emergency department attendance | Last record from 50% of organisations | 14 |
| 2023-09-01 | Emergency department attendance | Last record from 90% of organisations | 47 |
| 2023-09-01 | Emergency department attendance | Last record from 99% of organisations | 177 |
| 2023-09-01 | Inpatient admission | 50% of total records | 21 |
| 2023-09-01 | Inpatient admission | 90% of total records | 26 |
| 2023-09-01 | Inpatient admission | 99% of total records | 62 |
| 2023-09-01 | Inpatient admission | First record from 50% of organisations | 26 |
| 2023-09-01 | Inpatient admission | First record from 90% of organisations | 49 |
| 2023-09-01 | Inpatient admission | First record from 99% of organisations | 85 |
| 2023-09-01 | Inpatient admission | Last record from 50% of organisations | 47 |
| 2023-09-01 | Inpatient admission | Last record from 90% of organisations | 147 |
| 2023-09-01 | Inpatient admission | Last record from 99% of organisations | 197 |
| 2023-09-01 | Inpatient discharge | 50% of total records | 21 |
| 2023-09-01 | Inpatient discharge | 90% of total records | 28 |
| 2023-09-01 | Inpatient discharge | 99% of total records | 98 |
| 2023-09-01 | Inpatient discharge | First record from 50% of organisations | 26 |
| 2023-09-01 | Inpatient discharge | First record from 90% of organisations | 49 |
| 2023-09-01 | Inpatient discharge | First record from 99% of organisations | 85 |
| 2023-09-01 | Inpatient discharge | Last record from 50% of organisations | 47 |
| 2023-09-01 | Inpatient discharge | Last record from 90% of organisations | 160 |
| 2023-09-01 | Inpatient discharge | Last record from 99% of organisations | 201 |
| 2023-09-01 | Inpatient diagnosis codes | 50% of total records | 29 |
| 2023-09-01 | Inpatient diagnosis codes | 90% of total records | 64 |
| 2023-09-01 | Inpatient diagnosis codes | 99% of total records | 100 |
| 2023-09-01 | Inpatient diagnosis codes | First record from 50% of organisations | 26 |
| 2023-09-01 | Inpatient diagnosis codes | First record from 90% of organisations | 52 |
| 2023-09-01 | Inpatient diagnosis codes | First record from 99% of organisations | 99 |
| 2023-09-01 | Inpatient diagnosis codes | Last record from 50% of organisations | 49 |
| 2023-09-01 | Inpatient diagnosis codes | Last record from 90% of organisations | 161 |
| 2023-09-01 | Inpatient diagnosis codes | Last record from 99% of organisations | 202 |
